# A school-based food and nutrition education intervention increases nutrition-related knowledge and fruit consumption among primary school children in northern Ghana

**DOI:** 10.1186/s12889-024-19200-7

**Published:** 2024-06-29

**Authors:** Victor Mogre, Promise Emmanuel Sefogah, Alaofin Wemimo Adetunji, Oni Opeyemi Olalekan, Patience Kanyiri Gaa, Hannah N.G Ayettey Anie, Bamidele Tayo

**Affiliations:** 1 University for Development Studies, School of Medicine, Department of Health Professions Education and Innovative Learning, Tamale, Ghana; 2 University of Ghana Medical School, University of Ghana, Accra, Ghana; 3 Internal Medicine, University of Ilorin Teaching Hospital, Illorin, Nigeria; 4 Medical School, Ladoke Akintola University of Technology, Ogbomoso, Nigeria; 5 Department of Dietetics, University for Development Studies, Tamale, Ghana; 6 Department of Radiology, Korle-Bu Teaching Hospital, Accra, Ghana; 7 Parkinson School of Health Sciences, Loyola University, Chicago, IL, USA

**Keywords:** School-age, School-based food and nutrition education, Nutrition intervention, Diet, Competence

## Abstract

**Background:**

Providing children with the opportunity to learn about nutrition is critical in helping them establish a healthy lifestyle and eating behaviours that would remain with them till adulthood. We determined the effect of a school-based food and nutrition education (SFNE) intervention on the nutrition-related knowledge, attitudes, dietary habits, physical activity levels and the anthropometric indices (BMI-for-age z scores, %Body fat and waist circumference) of school-age children in northern Ghana.

**Methods:**

Following a controlled before-and-after study design, we recruited school-age children in primary 4 and 5 from public and private schools and assigned them non-randomly to intervention and control groups (4 schools total). A SFNE intervention called ‘Eat Healthy, Grow Healthy (EHGH)’ was implemented in intervention schools. Components of the intervention included children, teachers, school officials, and the school environment. Nutrition education didactic sessions, active discussions, nutrition games, charades, art work, and physical activity sessions were among the teaching and learning activities implemented. At 0 and 6 months, primary (anthropometry) and secondary (fruit, vegetable, and breakfast consumption) outcomes were obtained.

**Results:**

Mean BMI-for-age z-scores did not differ significantly between intervention and control groups (F_1,261_ = 0.45, *P* = 0.503, η^2^ = 0.01). However, significantly greater nutrition-related knowledge scores were recorded in the intervention group than in the control group at post-intervention (M = 6.07 SD = 2.17 vs. M = 5.22 SD = 1.92; *p* = 0.002). Mean number of days intervention children consumed fruits differed across time (F_1, 263_ = 33.04, *p* = 0.002, η^2^ = 0.04) but not between the control and intervention groups (F_1, 263_ = 0.28, *p* = 0.60, η^2^ = 0.00).

**Conclusions:**

The EHGH intervention had positive effects on the nutrition-related knowledge and the consumption of fruits among children although it did not impact their anthropometric indices.

## Introduction

Childhood excess weight has become a global public health concern, increasing in prevalence in both developed and developing countries [1]. In 2016, about 41 million under age 5 children were either overweight or obese and a quarter of which lived in Africa [2]. Over 340 million children and adolescents aged 5–19 years were overweight or obese in 2016 [2]. Although the prevalence of childhood overweight/obesity may be reducing in some developed countries it is on the rise in most low-and middle-income countries including those in sub-Saharan Africa [3]. In Ghana the prevalence of childhood overweight and obesity range between 10 and 21.2% and the prevalence is significantly higher among children in private schools [4, 5].

Excess body weight is a significant risk factor for diseases such as cardiovascular disease, type 2 diabetes and many cancers (e.g. colorectal cancer, kidney cancer and oesophageal cancer) [6, 7]. These diseases – often referred to as noncommunicable diseases (NCDs) – not only cause premature mortality, but also long-term morbidity [6, 7]. In addition, overweight and obesity in children are associated with adult obesity as well as reductions in quality of life [8, 9] and a greater risk of teasing, bullying and social isolation [10]. Providing children with the opportunity to learn about nutrition is critical in helping them establish a healthy lifestyle and eating behaviours that would remain with them till adulthood [11]. Dietary habits are developed at an early age, and children spend their formative years in school, thus providing an environment for healthy dietary habits to be formed if given the needed opportunities. The school setting is ideal for educating children early on about healthy lifestyles and healthy eating practices [12]. A recent systematic review has demonstrated that nutrition education interventions positively influence children’s energy intake, fruit and vegetable consumption, and sugar-sweetened drinks. It also promotes nutrition-related knowledge, values and practices [13]. The interventions were most effective when they offered opportunities for experiential learning and cross-curricula activities, connected with parents and were actively supported by teachers [14, 15]. The WHO Global Action Plan for non-communicable diseases [16], and the Rome Declaration on Nutrition and Framework for Action [17], have intensified calls and actions to create opportunities within the school environment to develop healthy dietary habits. It is possible to create such opportunities for children to learn about healthy eating alongside acquiring literacy skills. These calls and actions have influenced national educational policies to create opportunities for nutrition education in the school setting. Notwithstanding these, there is evidence to demonstrate that nutrition education policies have not yielded the desired outcomes in the school setting for various reasons, including underfunding and inadequate appropriate teaching and learning resources and the use of inappropriate teaching and learning strategies [18, 19]. In Ghana, many key barriers do not allow children to acquire healthy dietary and lifestyle habits during school, as evidenced by a recent study that explored barriers to implementing nutrition education in the basic school curriculum [20]. These barriers include inadequate nutrition education learning opportunities; lack of resources for practical, experiential delivery and active participation in nutrition education [20]. Furthermore, teachers’ inadequate knowledge of nutrition, the nonexistence of an explicitly stated policy on nutrition education and the non-involvement of parents and family members in nutrition education sessions are some of the teething challenges [20]. We evaluated the effect of a school-based food and nutrition education (SFNE) intervention on the nutrition-related knowledge, attitudes, dietary habits, physical activity levels and the anthropometric indices (BMI-for-age z scores, %Body fat and waist circumference) of school-age children in northern Ghana. We hypothesized that children from the intervention schools have improved nutrition-related knowledge, attitude, dietary habits, physical activity levels and reduced anthropometric indices.

## Methods

### Study design, participants, study setting and recruitment procedures

We employed a controlled before-and-after study design to evaluate the effects of a school-based food and nutrition education intervention on the nutrition-related knowledge, attitudes, anthropometric indices, dietary habits and physical activity levels of children. Based on evidence from a previous study [21] that evaluated children’s nutrition status, children were placed into intervention and control schools. We recruited school-age children in primary 4 and 5 aged 10–12 years from two private and two public schools across 4 suburbs in Tamale from July 2021 to January 2022. Participants were excluded if they had some form of disability that would prevent them from participating in vigorous physical activity and if they were boarders. Research assistants visited the schools for recruitment and data collection purposes. Permission was sought from the authorities of the selected schools, through the Heads/Principals of the school. Through the teachers the children were given informed consent forms for to be sent home to their parents to review and possibly sign. Children whose parents granted permission, provided informed consent and had assented to participate were included into the study. Ethical approval was granted by the Committee on Human Research, Publication and Ethics, Kwame Nkrumah University of Science and Technology (CHRPE/AP/120/21).

### Sample size determination

Clustering was done at the level of the school and we randomly picked two classes from each school resulting in 4 classes each for the intervention and control groups. Based on evidence from previous studies [22–24], we assumed differences between the two groups outcome (i.e. BMI for age z-scores) to be 0.25 BMI-for-age z-score with SD at 1.39 BMI-for-age z-scores, intra-cluster correlation coefficient (ICC) of 0.05, a rate of attrition of 10% [24] and power of 90% to yield a minimum sample size of 100 participants each for control and intervention group (a total of 200 participants). Given that we adopted a census approach at the school level, all children in the selected classes (i.e., primary 4 & 5) were included into the study to yield a sample size of 299 at baseline.

### Intervention

Children in the intervention schools received an intervention called ‘Eat Healthy, Grow Healthy’ (EHGH), which was designed to improve the anthropometric indices and dietary and lifestyle habits of the children. Children in the control schools followed their usual health and physical education curriculum with no intervention programme.

The EHGH intervention followed a multicomponent approach involving children, teachers, school authorities and parents. The intervention was informed by the socio-ecological model covering the children, family and the school environment. It was also designed to fit into the WHO/FAO tripartite approach to nutrition education involving the family, community and the school covering the classroom, and the school environment [25].

**Fig. 1:**
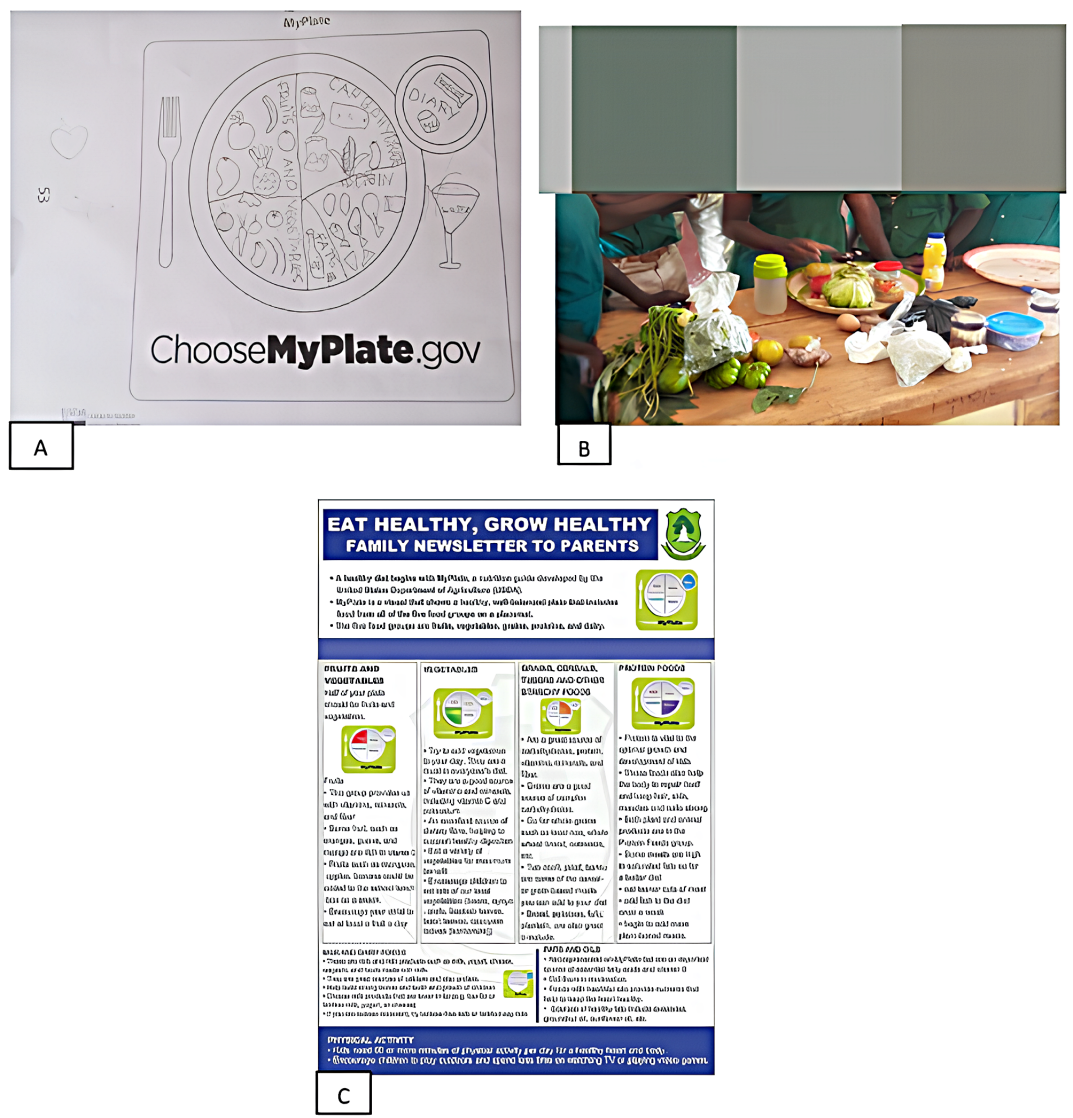
Children’s drawing of the food groups using MyPlate (**A**); Children practical demonstrations of MyPlate using food items (**B**) and family newsletter connecting parents to the intervention (**C**)

At the school level, we adopted nutrition education, physical activity education, enabling healthy school environment and strengthening school policies on healthy diet. To link up with the family, we designed family newsletters to enable what is learned in the classroom shared with the parents of the children. We visited the intervention schools weekly for 6 consecutive weeks to administer the intervention. Each session lasted for 60 min with break between sessions. To ensure active participation by the children, we included indoor (e.g., nutrition information, reading aloud, active discussions, nutrition games, charades, and artwork) and outdoor activities (e.g., exercises). Children also watched videos that introduced them to nutrition concepts before every didactic and practical session. Healthy snacks such as fruits were shared to the children at the end of each session. Additional initiatives to foster a healthy school environment were establishing school nutrition clubs and engaging school authorities and vendors to promote the sale of fruits and reduce the availability of candies and sugary beverages on school grounds. The design, course content and activities of the intervention were adapted from previous publications including the University of California Shaping Healthy Choices Program [26, 27], and the Food and Agriculture Organisation Nutrition Education for Primary Schools Volumes 1 and 2 [25]. The course content included topics such as the sources and origins of foods, the components of a healthy diet, dietary requirements, essential nutrients, MyPlate guidelines, food labelling, and promoting physical activity. To encourage active participation from the children during the sessions, various literary activities, such as drawing and visualizing the MyPlate, were conducted. Common food items were brought to the sessions to demonstrate to children how to read nutrition fact labels on foods (Shown in Fig. 1).

The food traffic system was also used to demonstrate to children on how to make healthy food choices. Colourful posters were also designed and posted in the classrooms of the children. Three family newsletters were created and distributed to children to be given to their parents to establish a connection with the family. The newsletters covered topics such as using the MyPlate as a guide for healthy eating, being a healthy role model for your children, and the food traffic system. The goal of these newsletters was to raise awareness about nutrition and enable parents to support their children to eat healthy foods and also serve as role models for their children.

### Outcomes and measures

**BMI-for-age Z-scores**: Anthropometric measurements of weight and height of the children were measured at baseline and at 6 months. The weight of the children was measured using a bioimpedance body composition monitor manufactured by Tanita (Model: BC-587), that has an option for measuring weight. Height was measured using seca 217 stable stadiometer. In both measurements, children were asked to wear light clothing with their shoes removed. These values were used to generate age- and sex-specific standard deviation scores using the WHO Body-Mass-Index-for-age Z-scores by means of the WHO Anthroplus software for 5-19-year-old. Using the WHO classifications, the following criteria were used: severe thinness < − 3 Z score, normal weight < + 1 Z score, to < -2 Z score, overweight > 1 Z score and obesity > 2 Z score from the mean value of the reference population.

**Percentage body fat**: Was measured using a bioimpedance body composition monitor manufactured by Tanita (Model: BC-587).

**Waist circumference**: Was measured using a flexible tailor’s tape measure to the nearest centimetre while children were in a standing position at the end of a gentle expiration. The anatomical landmarks used were: laterally, midway between the lowest portion of the rib cage and iliac crest and anteriorly, midway between the xiphoid process of the sternum and umbilicus [28].

### Secondary outcomes

#### Nutrition-related knowledge and attitude;

Nutrition-related knowledge was assessed using 10 items derived from previous studies [26, 27], comprising largely test questions from the University of California Shaping Healthy Choices Program that have been validated and found reliable to assess nutrition knowledge in children. A wrong answer attracted zero (0) score and a correct answer was scored one (1) to yield a maximum score of 10. The questions were related to food nutrients, sources and origins of foods, constituents of a healthy diet, food labels and MyPlate. Attitude towards nutrition was assessed using a 14-item questionnaire. Using a 5-point Likert scale, children were asked to indicate their level of agreement to a list of statements ranging from 1-strongly disagree to 5-strongly agree. Total scores were generated and weighted to range between 1 and 5. The items were derived from previously validated questionnaires [29].

#### Physical activity levels;

Was assessed using the physical activity questionnaire for children (PAQ-C). The PAQ-C is a recall 10-item questionnaire that evaluates the physical activity levels of children during the last 7 days. The previously validate PAQ-C, measures different physical activities during physical education classes, lunch break, recess, after school, in the evenings and during weekends [30].

**Dietary habits**: This was determined by measuring the consumption of vegetables, fruits and breakfast using items from a previous validated study from Ghana [31]. Consumption of vegetables was evaluated by the question: ‘During the past 7 days on how many days did you usually eat vegetables such as kontomire, garden eggs, lettuce, cabbage, okra, alefu, bra, ayoyo, or bean leaves?’. Fruit consumption was assessed by: ‘During the past 7 days, on how many days did you usually eat fruit, such as oranges, pineapple, watermelon, banana, guava, pear, apple, mangoes, or pawpaw?’. Breakfast consumption was measured using the question: ‘During the past 7 days on how many days did you usually eat breakfast?’. In all of these questions the responses ranged from 0 to 7 days.

### Statistical analysis

Differences between the intervention and control groups on socio-demographic characteristics and the primary and secondary outcomes of the children at baseline and at follow up were compared using Student’s *t*-test, ANOVA and Chi-square tests. Repeated-measures ANOVAs were used to test for differences between the control and intervention group (between-subjects factor) for both primary and secondary outcomes at the two time-points (within-subjects factor). All analysis were based on the intention-to-treat principle. SPSS version 20.0 was used for data analysis. Significance level was set at *P* < 0.05.

## Results

### General characteristics of the participants

**Fig. 2:**
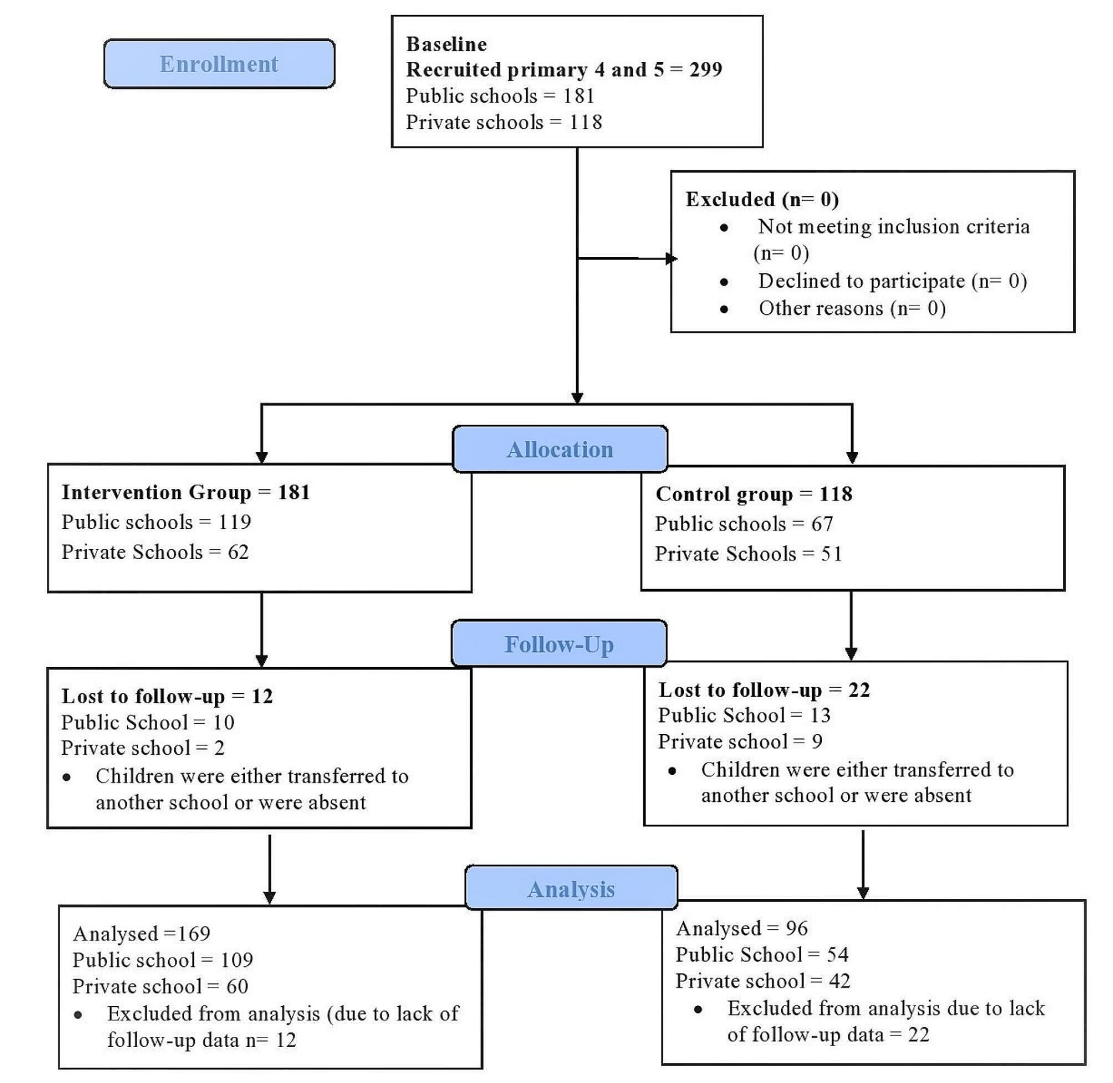
Flow chart showing recruitment and follow-up

We recruited 299 children at baseline in which 181 were in the intervention group and 118 in the control group (Shown in Fig. 2). At post-intervention, 34 students were lost to follow due to either school transfer or absence (i.e., 12 children in the intervention schools and 22 in the control schools).

The loss to follow children had a mean age of 12.09 ± 2.52 years, 21 females, mean %BF of 17.74 ± 10.44, five were overweight/obese (i.e., 3 from the control schools and 2 from the intervention school) and three were thin (i.e., 1 from the control schools and 2 from the intervention schools). Their data were eliminated, and we included the 265 children that had complete data (169 for intervention and 96 for control group). As shown in Table 1, slightly over 50% of the participants were male children, 61.5% attended public schools (64.5% intervention schools and 56.3% control schools), and 53.2% were in primary 4. At baseline the mean age was 11.34 ± 1.47 years and mean number of siblings was 4.78 ± 2.70. Both intervention and control groups did not differ significantly by gender (Female = 85(50.3%) vs. 43(55.2%); *p* = 0.443) and age (11.20 ± 1.27 vs. 11.58 ± 1.75; *p* = 0.059) at baseline but differed by number of siblings (5.12 ± 2.94 vs. 4.17 ± 2.10; *p* = 0.002). At 6 months post -intervention, age (11.88 ± 1.50 vs. 12.32 ± 2.01; *p* = 0.062) and number of siblings (4.03 ± 2.16 vs. 3.72 ± 1.67; *p* = 0.315) did not differ significantly between control and intervention groups.

**Table 1:** General characteristics of the children at baseline between intervention and control groups

Variable	Intervention (*n* = 169)	Control (*n* = 96)	Total (*n* = 265)
Baseline			
Gender			
Female	85(50.3%)	43(44.8%)	128(48.3%)
Male	84(49.7%)	53(55.2%)	137(51.7%)
Age, Mean (SD)	11.20 ± 1.27	11.58 ± 1.75	11.34 ± 1.47
**Class**			
Primary 4	83(49.1%)	61(63.5%)	144(54.3%)
Primary 5	86(50.9%)	35(36.5%)	121(45.7%)
**Status of school**			
Public	109(64.5%)	54(56.3%)	163(61.5%)
Private	60(35.5%)	42(43.8%)	102(38.5%)
Mean number of Siblings	5.12 ± 2.94	4.17 ± 2.10	4.78 ± 2.70

Table 2 shows the difference in mean scores for both primary and secondary outcomes between baseline and post-intervention and between the two groups. In both intervention and control groups, the mean BMI-for-age z-scores did not differ significantly at baseline and at 6 months. The prevalence of thinness/severe thinness decreased by 2.40% among intervention children after following the intervention (i.e., from 9.5%, *n* = 16 at baseline to 7.1%, *n* = 12 at post-intervention) but did not change in the control group (i.e. 7.4%, *n* = 7 for both time points) (*p* = 0.561). At 6 months post-intervention, the prevalence of normal weight status increased by 3% in the intervention group (i.e., 72.6%, *n* = 122 at baseline to 75.6%, *n* = 127 at 6 months) but remained same in the control group at 72.6% (*n* = 71) for both time points (*p* = 0.727). The differences were not statistically significant. At 6 months, the prevalence of overweight/obesity decreased by 0.6% in the intervention group (i.e., 17.9%, *n* = 30 at baseline to 17.3%, *n* = 29 at 6 months) but did not change in the control group for both time points remaining at 17.9% (*n* = 17) (*p* = 1.000). At baseline, control children (66.64 ± 8.55) had statistically significant (*p* = 0.010) higher mean (SD) waist circumferences than intervention children (63.99 ± 7.88). However, after 6 months, there were no significant differences between the two groups, although the control children had higher values (68.67 ± 11.32 vs. 67.19 ± 7.40; *p* = 0.199). Both intervention and control groups recorded increased mean waist circumferences compared to the baseline values. The increment in both groups were not statistically significant (*p* = 0.199). At 6 months, the number of days intervention children ate fruits in a week were significantly higher than the number of days the control children ate fruits (3.82 ± 2.16 vs. 3.74 ± 2.13). At baseline, there were no significant differences in the number of days the children ate fruits for both intervention and control groups. The mean nutrition-related knowledge scores of the intervention children increased significantly (*p* < 0.001) from 4.78 ± 0.59 at baseline to 6.07 ± 2.17 at 6 months. Although the control children also recorded increased nutrition-related knowledge scores between baseline (4.89 ± 1.38) and at 6 months (5.22 ± 1.92), the differences were not statistically significant (*p* = 0.084). At baseline, the intervention and control children did not differ significantly in their mean nutrition-related knowledge scores.

**Table 2:** Mean values of primary and secondary outcomes at baseline and at 6 months

Primary outcomes		Baseline		Post			
Variable	Group	Mean (SD)	*N*	Mean (SD)	*n*	Change (95%CI)	*p*-value
**% Body fat**	Intervention	15.95(8.19)	168	16.26(8.34)	168	0.31 (-0.24–0.860	0.272
	Control	16.59(7.79)	96	16.33(7.77)	96	-0.26 (-1.08–0.55)	0.525
Mean difference		-0.64(-2.67–1.39)		-0.07 (-2.11–1.98)			
**Waist circumference**	Intervention	63.99(7.88)	158	67.19(7.40)	158	3.19 (2.59–3.80)	**< 0.001**
	Control	66.64(8.55)	106	68.67(11.32)	106	2.03 (0.33–3.73)	**0.020**
Mean difference		-2.63 (-4.66 - -0.63) *		-1.48 (-3.75–0.79)			
**BAZ**	Intervention	-0.33(1.34)	168	-0.26(1.46)	168	0.07 (-0.02–0.17)	0.141
Mean difference	Control	-0.23(1.33)	95	-0.21(1.32)	95	0.02 (-0.05–0.10)	0.517
Mean difference		-0.10(-0.45–0.25)		-0.05 (-0.41–0.31)			
**Secondary outcomes**							
Number of days fruits are eaten in a week	Intervention	3.10(2.20)	169	3.82(2.16)	169	0.72 (0.32–1.11)	**< 0.001**
	Control	3.42(2.37)	96	3.74(2.13)	96	0.32 (-0.21–0.86)	0.238
Mean difference		-0.32(-0.25–0.89)		0.08 (-0.46–0.62)			
Number of days vegetables are eaten in a week	Intervention	3.72(2.23)	163	3.89(2.19)	163	0.166 (-0.22–0.55)	0.401
	Control	4.00(2.31)	94	3.69(2.04)	94	-0.31 (-0.92–0.30)	0.319
Mean difference		-0.28(-0.85- 0.30)		0.20 (-0.35–0.74)			
Number of days breakfast is eaten/week	Intervention	5.65(2.28)	168	5.35(2.40)	168	-0.31 (-0.77–0.15)	0.186
	Control	5.58(2.53)	95	5.41(2.22)	95	-0.17 (-0.75–0.41)	0.566
Mean difference		0.08(-0.52–0.68)		-0.07 (-0.66–0.53)			
Attitude scores	Intervention	3.93(0.64)	167	3.98(0.68)	167	0.05 (-0.08–0.18)	0.416
	Control	3.98(0.75)	93	3.83(0.65)	93	-0.16 (-0.37–0.06)	0.150
Mean difference		-0.05(-0.23–0.12)		0.16 (-0.02–0.33)			
Nutrition-related Knowledge scores	Intervention	4.78(0.59)	169	6.07(2.17)	169	1.29 (0.96–1.63)	**< 0.001**
	Control	4.89(1.38)	96	5.22(1.92)	96	0.33 (-0.04–0.71)	0.084
Mean difference		-0.10(-0.54–0.33)		0.85 (0.33–1.38) *			
Physical activity levels	Intervention	2.48(0.63)	169	2.38(0.59)	169	-0.10 (-0.23–0.03)	0.139
	Control	2.25(0.63)	96	2.29(0.65)	96	0.03 (-0.14–0.20)	0.689
Mean difference		0.23(0.07–0.38) *		0.09 (-0.06–0.25)			

*p-value < 0.05

Repeated-measures ANOVA (shown in Table 3) showed a statistically non-significant difference for time (BAZ scores before and after intervention; *F*_1 ,261_ = 1.86, *P* = 0.173, η^2^ = 0.01), group alone (*F*_1 ,261_ = 0.18, *P* = 0.669, η^2^ = 0.00) and group (intervention vs. control) by time interaction (*F*_1,261_ = 0.45, *P* = 0.503, η^2^ = 0.01). Similar results were found for % body fat. Regards waist circumference, statistically significant difference was found across time (F_1, 262_ = 43.19, *p* < 0.001, η^2^ = 0.14 and group alone (F_1, 262_ = 4.14, *p* = 0.043, η^2^ = 0.02) but not group by time interaction (F_1, 262_ = 2.13, *p* = 0.146, η^2^ = 0.01). Also, the repeated measures ANOVA showed a statistically significant difference across time for the consumption of fruits (F_1, 263_ = 33.04, *p* = 0.002, η^2^ = 0.04) but not for group alone (F_1, 263_ = 0.28, *p* = 0.60, η^2^ = 0.00) and group by time interaction (F_1, 263_ = 4.73, *p* = 0.243, η^2^ = 0.01).

**Table 3:** Intervention effects (time by group interaction) on primary and secondary outcomes

	Time			Group			Time*Group		
Variable/group	F (df)	*P*	ηp2	F(df)	*p*	ηp2	F(df)	*p*	ηp2
BAZ	1.86(1, 261)	0.173	0.01	0.18(1, 261)	0.669	0.00	0.45(1, 261)	0.503	0.01
% Body fat	0.01(1, 262)	0.921	0.00	4.14(1, 262)	0.726	0.00	1.40 (1, 262)	0.239	0.01
Waist circumference	43.19(1, 262)	< 0.001	0.14	4.14(1, 262)	0.043	0.02	2.13(1, 262)	0.146	0.01
Number of days fruits are eaten in a week	33.04(1, 263)	0.002	0.04	0.28(1, 263)	0.60	0.00	4.73(1, 262)	0.243	0.01
Number of days vegetables are eaten in a week	0.17(1, 255)	0.683	0.00	0.03(1, 255)	0.862	0.00	1.85(1, 255)	0.175	0.01
Number of days breakfast is eaten/week	1.58(1, 261)	0.210	0.01	0.001(1,261)	0.982	0.00	0.14(1,261)	0.711	0.00
Nutrition-related attitude	0.77(1, 258)	0.383	0.00	0.61(1,258)	0.435	0.00	3.12(1, 258)	0.78	0.01
Nutrition-related Knowledge scores	36.95(1, 263)	> 0.001	0.12	3.31(1, 263)	0.070	0.01	28.01(1, 263)	< 0.001	0.05
Physical activity levels	0.34(1, 263)	0.562	0.00	7.54(1, 263)	0.006	0.03	1.48(1, 263)	0.225	0.01

**Fig. 3:**
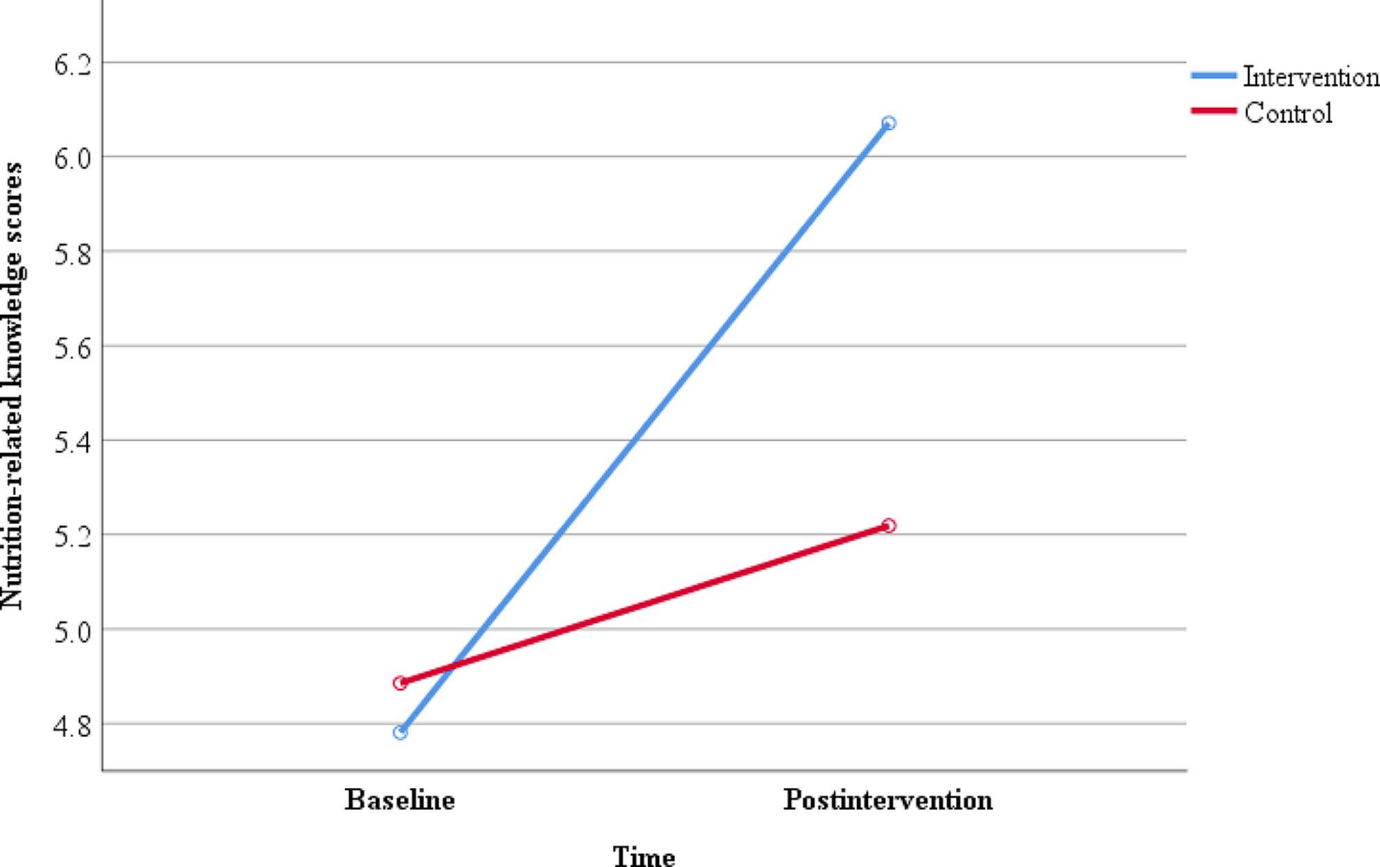
Nutrition-related knowledge scores between groups at baseline and postintervention

Regards nutrition-related knowledge, there was a statistically significant difference between the two time points i.e., baseline and post-intervention (F_1, 263_ = 36.95, *p* < 0.001 η^2^ = 0.12) and group by time interaction (F_1, 263_ = 28.01, *p* < 0.001, η^2^ = 0.05) but not group alone (F_1, 263_ = 3.31, *p* = 0.070, η^2^ = 0.01). Post hoc comparisons using the Bonferroni adjustment showed that both groups had almost similar scores at baseline (M = 4.78 SD = 0.59 vs. M = 4.89 SD = 1.38 *p* = 0.639) but significantly greater scores were recorded in the intervention group than in the control group at post-intervention (M = 6.07 SD = 2.17 vs. M = 5.22 SD = 1.92; *p* = 0.002) (Shown in Fig. 3).

## Discussion

The intervention significantly improved the nutrition-related knowledge of children in the intervention group and the number of days they ate fruits in a week. However, the BMI-for-age z-scores, and %body fat remained unchanged in both intervention and control groups except for waist circumference that recorded positive improvement at post-intervention.

Similar to those of previous studies in Ghana [32, 33] and North India [34] we did not find significant differences in the BMI-for-age z-scores and %BF between the intervention and control groups at post-intervention. However, there were significant increases in the waist circumference of the children in both intervention and control groups at post-intervention. There were also positive increases in the BMI and %BF of the children for both groups but the differences were not significant. As reported in a previous study [33] from Ghana, the positive changes could be due to the age of the children being at the pubertal stage which is a period of growth spurt characterized by increase in muscle mass, body weight, etc. The impact of SFNE interventions on preventing weight gain or obesity is varied as a recent systematic review of 39 studies found 14 studies reporting reduction in BMI/BMI-for-age z-scores and concluded that SFNE may have the potential to effectively reduce the BMI/BMI z-scores of adolescents to a healthier range [35]. However, a Cochrane review of 19 SFNE interventions found that SFNE could promote a healthy diet and increase physical activity levels, but not effective in preventing weight gain [36]. The duration of the intervention might have contributed to the insignificant impact of the intervention on body weight of the children as there is evidence that SFNE interventions with longer durations and high intensity of over a year are more likely to be effective than those with shorter duration [35, 36]. A 3-year obesity intervention in school children in Beijing found the prevalence of overweight and obesity reducing by 26.3% and 32.5% respectively in intervention schools respectively after intervention while the prevalence of obesity increased in the control schools [37].

The intervention had positive impacts on children who were thin as we found that the proportion of children that were thin decreased at post-intervention in the intervention group although the differences were not statistically significant. Malnutrition in children such as thinness, stunting and underweight is endemic in the study area and this intervention demonstrates that school-based nutrition education that involves parents may relatively be an important medium through which malnutrition in children could be tackled.

We found a significant positive change in nutrition-related knowledge of children in the intervention group. This is similar to previous studies conducted in Ghana [32, 33] as well as in North India and the UK [34, 38]. Another important finding of this study was that the intervention improved the number of days children consumed fruits in a day but not vegetables. This is similar to a study conducted in India in which Singhal et al. [34] reported a positive significant change in the consumption of fruits among adolescents in the intervention group compared to the control groups. Our findings were however contrary to those reported among Ghanaian children by Antwi et al. [32] who did not find any significant differences in the dietary diversity scores between intervention and control children post-intervention. In another study from Ghana among overweight and obese children, Addo et al. [33] did not find significant changes in the practice of healthy dietary habits between the control and intervention groups post-intervention. The differences in findings could be due to variations in measurements as the studies reported by Addo et al. [33] and Antwi [32] did not measure the consumption of fruits or vegetables separately making it difficult to determine the effect of the nutrition education interventions on these separate dietary habits or behaviours.

It is worth noting that the intervention is promising due to our adoption of child-centred learning strategies that were blended with age-appropriate pedagogy (such as interactive classroom sessions, online sessions, demonstrations, charades, nutrition games, drawing and colouring the eat well plate). This provided children with authentic, differentiated experiences that captured their interests and imaginations [39]. Our connection with parents through family newsletters probably helped to create awareness about what the children were learning in school that led to parents supporting their children to improve their dietary habits and probably parents own dietary habits, thereby improving the dietary habits of the entire family. Connecting with parents could present another approach through which SFNE could be used as a channel to improve parents’ nutrition-related attitudes and practices.

The intervention did not significantly impact on the consumption of vegetables, breakfast consumption, attitudes and physical activity levels of the children. The adoption of healthy dietary habits such as these is not only influenced by knowledge and attitudes but other factors such as food availability, accessibility, affordability, income levels, etc., that are beyond the control of the children. Future interventions should be considered to support children and parents to overcome these barriers.

The evidence from this study demonstrates that it is possible to carry out a school-based intervention, providing children with opportunities to learn about nutrition that could potentially shape their dietary and lifestyle habits. The eagerness and the enthusiasm of the intervention children to actively participate in the activities was something to behold. We thus believe the intervention was more effective in improving several other psychomotive behaviours of the children we probably did not measure.

The intervention had some limitations. The non-blinded, non-random allocation of the schools has a potential for bias at the level of the school. The intervention did not cover many schools, affecting the generalizability of the findings. However, the findings have some level of external validity and can be used as a basis to design future interventions and scalable as a public health program in Ghana. There were also minimal chances of contamination as the intervention and control schools were located at a reasonably large distance from each other. Another limitation of the study was our inability to achieve the minimum sample size for one of the classes in the control schools. However, the differences were compensated by the high number from the other control classes. Furthermore, the statistical analysis was compared between the control and intervention groups, but not between individual classes.

## Conclusion

Although the EHGH intervention did not have significant impact on the BMI-for-age z-scores, % BF and WC of the children, it demonstrated positive impact on the nutrition-related knowledge and the adoption of healthy dietary habits such as the consumption of fruits by the children. In addition, the intervention could also be used to reduce undernutrition among school-age children as it reduced the prevalence of thinness among intervention children.

## Data Availability

Data is available upon request from the corresponding author.
